# Supplementary material for: Structural basis of neurosteroid anesthetic action on GABAA receptors
Source: Nat Commun. 2018 Sep 28;9:3972. doi: 10.1038/s41467-018-06361-4 (PMC6162318; doi:10.1038/s41467-018-06361-4)
Supplement: Supplementary file 1 — Supplementary Information [file 41467_2018_6361_MOESM1_ESM.pdf]

Structural Basis of Neurosteroid Anesthetic Action on $GABA_A$ Receptors

Q. Chen et al.

```
                                                  . . * .:*...**:..:
ELIC              ---------------------------APADNAADARPVDVSVSIFINKI    23
α1GABAAR          QPSLQDELKDNTTVFTRILDRLLDGYDNRLRPGLGERVTEVKTDIFVTSF    50
ELIC-α1GABAAR     ---------------------------APADNAADARPVDVSVSIFINKI    23

                    *.  :  *.:* ::  .*..:  *  *  .::     ::. : . :*.
ELIC              YGVNTLEQTYKVDGYIVAQWTGKPRKTPGDKPLIVENTQIERWINNGLWV    73
α1GABAAR          GPVSDHDMEYTIDVFFRQSWKDERLKFKGPMTVLR----LNNLMASKIWT    96
ELIC-α1GABAAR     YGVNTLEQTYKVDGYIVAQWTGKPRKTPGDKPLIVENTQIERWINNGLWV    73

                  *   * *   *         ** * :  ** ::*. *:     .  *.:. *
ELIC              PALEFINVVGS----PDTGNKRLMLFPDGRVIYNARFLGSFSNDMDFRLF   119
α1GABAAR          PDTFFHNGKKSVAHNMTMPNKLLRITEDGTLLYTMRLTVRAECPMHLEDF   146
ELIC-α1GABAAR     PALEFINVVGS----PDTGNKRLMLFPDGRVIYNARFLGSFSNDMDFRLF   119

                  *:* :   *:: .::*.. :: :.  :  :..     *...:::: : *:
ELIC              PFDRQQFVLELEPFSYNNQQLRFSDIQVYTEN----IDNEEIDEWWIRGK   165
α1GABAAR          PMDAHACPLKFGSYAYTRAEVVYEWTREPARSVVVAEDGSRLNQYDLLGQ   196
ELIC-α1GABAAR     PFDRQQFVLELEPFSYNNQQLRFSDIQVYTEN----IDNEEIDEWWIRGK   165

                  :            . **.. .*:  :*.::.  *: .*:: .  **  :
ELIC              ASTHISDIRYDHLSSVQPNQNEFSRITVRIDAVRNPSYYLWSFILPLGLI   215
α1GABAAR          TVD---------SGIVQSSTGEYVVMTTHFHLKRKIGYFVIQTYLPCIMT   237
ELIC-α1GABAAR     ASTHISDIRYDHLSSVQPNQNEFSRITVRIDAVRKIGYFVIQTYLPCIMT   237
                                                           TM1
                  :  *   ***  **.. *   ..* :**:.: :: : * **::.*:*.:*
ELIC              IAASWSVFWL--ESFSERLQTSFTLMLTVVAYAFYTSNILPRLPYTTVID   263
α1GABAAR          VILSQVSFWLNRESVPARTVFGVTTVLTMTTLSISARNSLPKVAYATAMD   287
ELIC-α1GABAAR     VILSQVSFWLNRESVPARTVFGVTTVLTMTTLSISARNSLPKVAYATAMD   287
                                           TM2
                   :* . *. :*:*:: :  .::   .*      .    *:*       ::.
ELIC              QMIIAGYGSIFAAILLIIFAHHRQANGVE---DDLLIQRCRLAFPLGFLA   310
α1GABAAR          WFIAVCYAFVFSALIEFATVNYFTKRGYA / NSVSKIDRLSRIAFPLLFG   405
ELIC-α1GABAAR     WFIAVCYAFVFSALIEFATVNYFTKRGVE---SVSKIDRLSRIAFPLLFG   405
                       TM3                                     TM4
                  *  ::
ELIC              IGCVLVIRGITL-----------    322
α1GABAAR          IFNLVYWATYLNREPQLKAPTPHQ   429
ELIC-α1GABAAR     IFNLVYWATYLN------------   417
```

Supplementary Figure 1. Construction of a functional ELIC-α1GABAAR chimera. The α1GABAAR chimera was built by merging the extracellular domain of ELIC (yellow, A1 to R199) and the transmembrane domain of human α1GABAAR (orange, K222 to N417). To facilitate crystallization, the large intracellular loop between TM3 and TM4 in α1GABAAR (G313 to N387, not shown) was replaced by the tripeptide linker from ELIC (GVE) and 12 residues at the C-terminus of α1GABAAR (R418 to Q429) were removed. The underlined residues participate in the α-helical structures in the TMD.

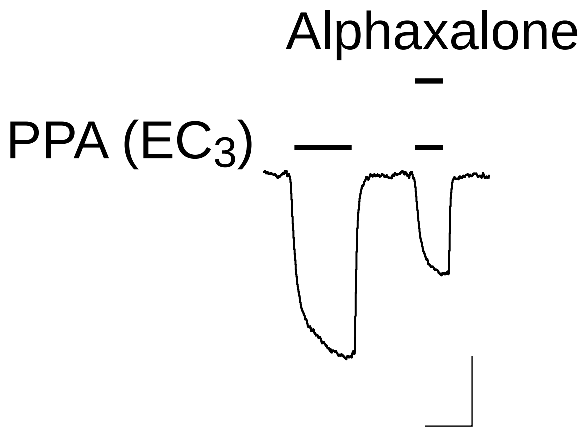

Supplementary Figure 2. Alphaxalone inhibits ELIC. A representative trace showing that ELIC is inhibited by alphaxalone (10 μM). Black bars over the trace indicate agonist and drug application times. The vertical and horizontal scale bars represent 50 nA and 30 seconds, respectively. The inhibition of ELIC by alphaxalone is in contrast to the alphaxalone potentiation and activation of ELIC-α1GABA$_A$R shown in Fig. 1 in the main text.

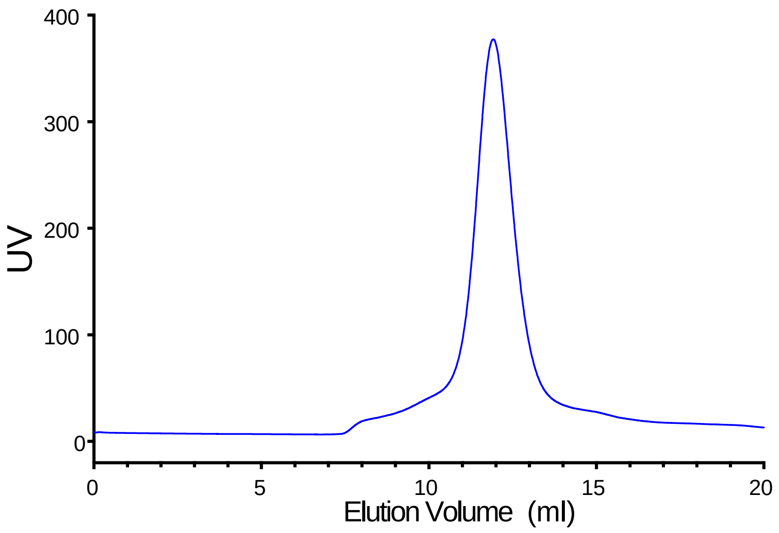

Supplementary Figure 3. Size exclusion chromatography purification of ELIC-α1GABA$_A$R (blue) in DDM, showing a peak eluded at a volume consistent with the size of a pentameric α1GABA$_A$R chimera. The purified protein is suitable for crystallization.

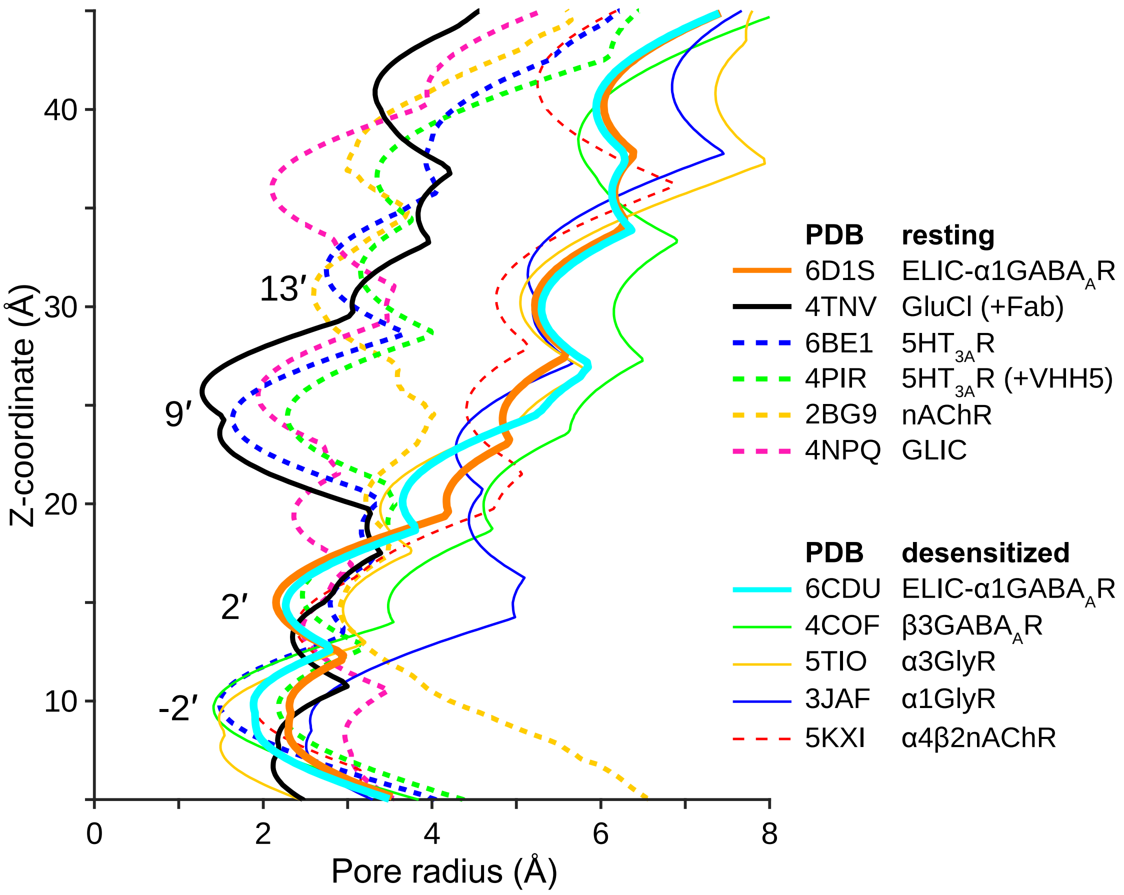

Supplementary Figure 4. Pore profile comparison of the α1GABAAR chimeras with representative resting and desensitized pLGICs. The resting state channels include: GluCl[1], 5HT3AR[2,3], nAChR[4], and GLIC[5]. The desensitized channels include: β3GABAAR[6], α3GlyR[7], α1GlyR[8], and α4β2nAChR[9]. The pore profiles for anion channels are shown with a solid line and for cation channels with a dashed line. For the resting state structures, the most constricted pore radius is at the 9′ position in 5HT3AR (PDB: 6BE1)[2] and GluCl (PDB: 4TNV)[1]; at the -1′ position in the crystal structure of 5HT3AR (PDB: 4PIR)[3]; at the 13′ position in nAChR[4]; and at the 2′ position in the crystal structure of our α1GABAAR chimera. For all the desensitized structures, including our α1GABAAR chimera desensitized by alphaxalone, the most constricted pore radius occurs exclusively at the -2′ position. The pore profile comparison with ELIC[10], GLIC-α1GABAAR[11], β3-α5GABAAR[12], and β3GABAAR[6] are shown in Fig. 3c in the main text.

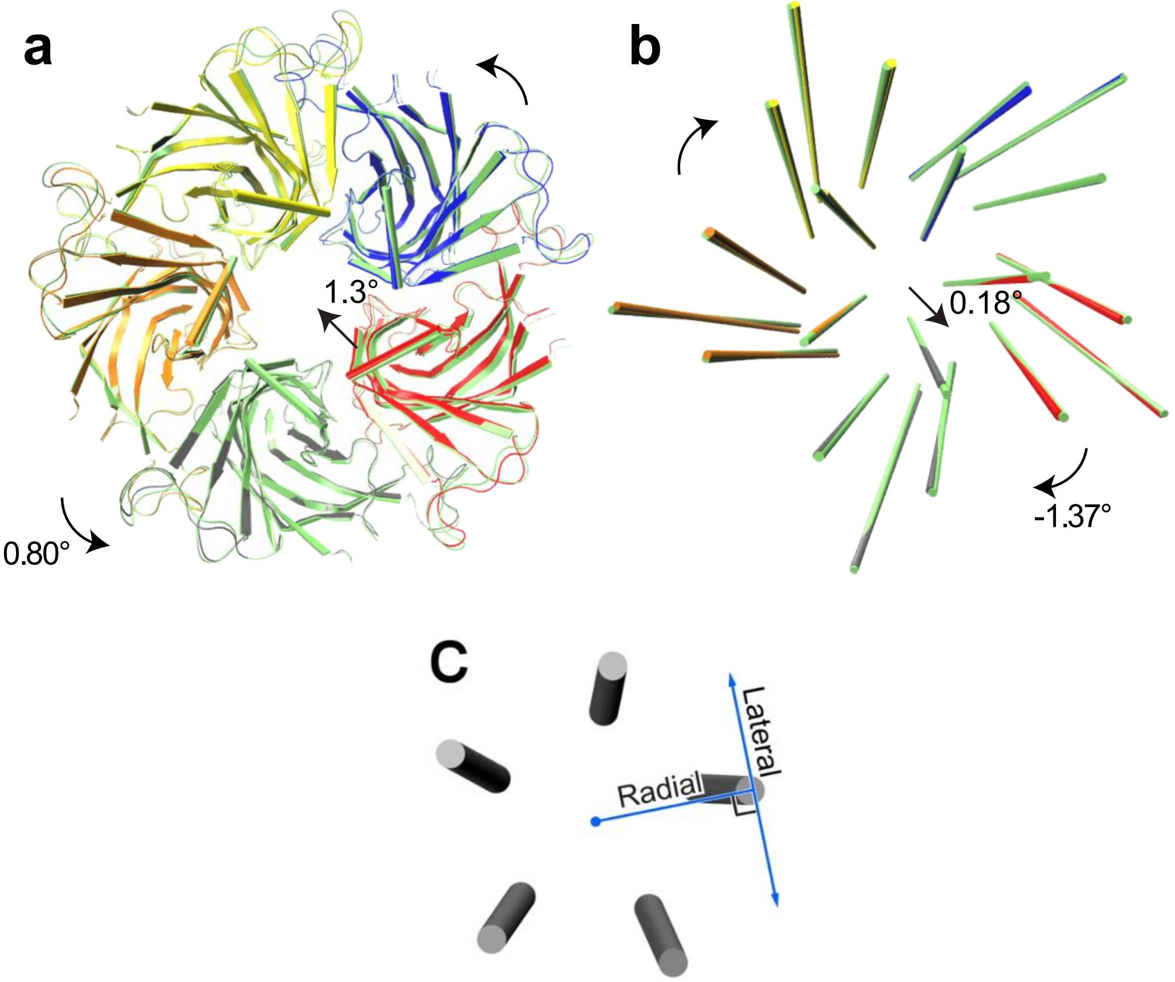

Supplementary Figure 5. **Small quaternary structural changes in comparison of the apo and alphaxalone-bound ELIC-α1GABA$_A$R**. (**a**) The ECD of the alphaxalone-bound α1GABA$_A$R chimera (multi-colored) rotates (0.80°) laterally counter-clockwise compared to the apo α1GABA$_A$R chimera (green), while (**b**) the TMD of the alphaxalone-bound α1GABA$_A$R chimera (multi-colored) rotates laterally (-1.37°) clockwise compared to the apo α1GABA$_A$R chimera (green), in the opposite direction of the ECD displacement. Counter-clockwise twisting of the ECD in pLGICs is associated with channel activation. Relative to the apo α1GABA$_A$R chimera, insignificant radial change (blooming) is observed in both the ECD (1.3° inward) and TMD (0.18° outward) of the alphaxalone-bound α1GABA$_A$R. These results are consistent with the fact that both channels are closed channels. **(c)** Lateral and radial angles used to determine twisting and blooming are defined as reported previously[1,5,13,14,15].

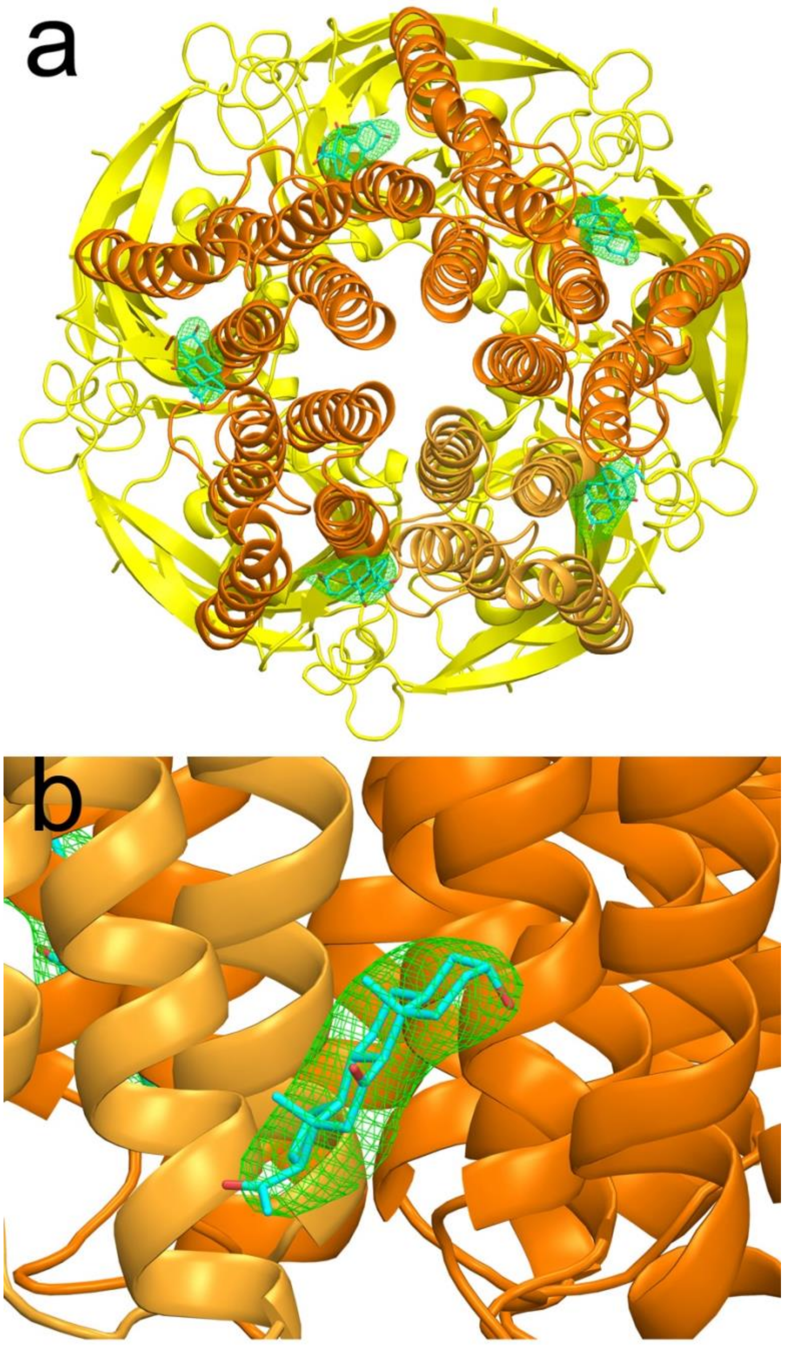

Supplementary Figure 6a. Bottom and side views of alphaxalone binding to the α1GABA$_A$R chimera. Alphaxalone (molecule in cyan color) binding to the inter-subunit sites in the TMD is indicated by the F_O-F_C omit electron density map (green mesh) contoured at 3σ. The alphaxalone fitting to the 2F_O-F_C electron density map is shown in Fig. 4b in the main text.

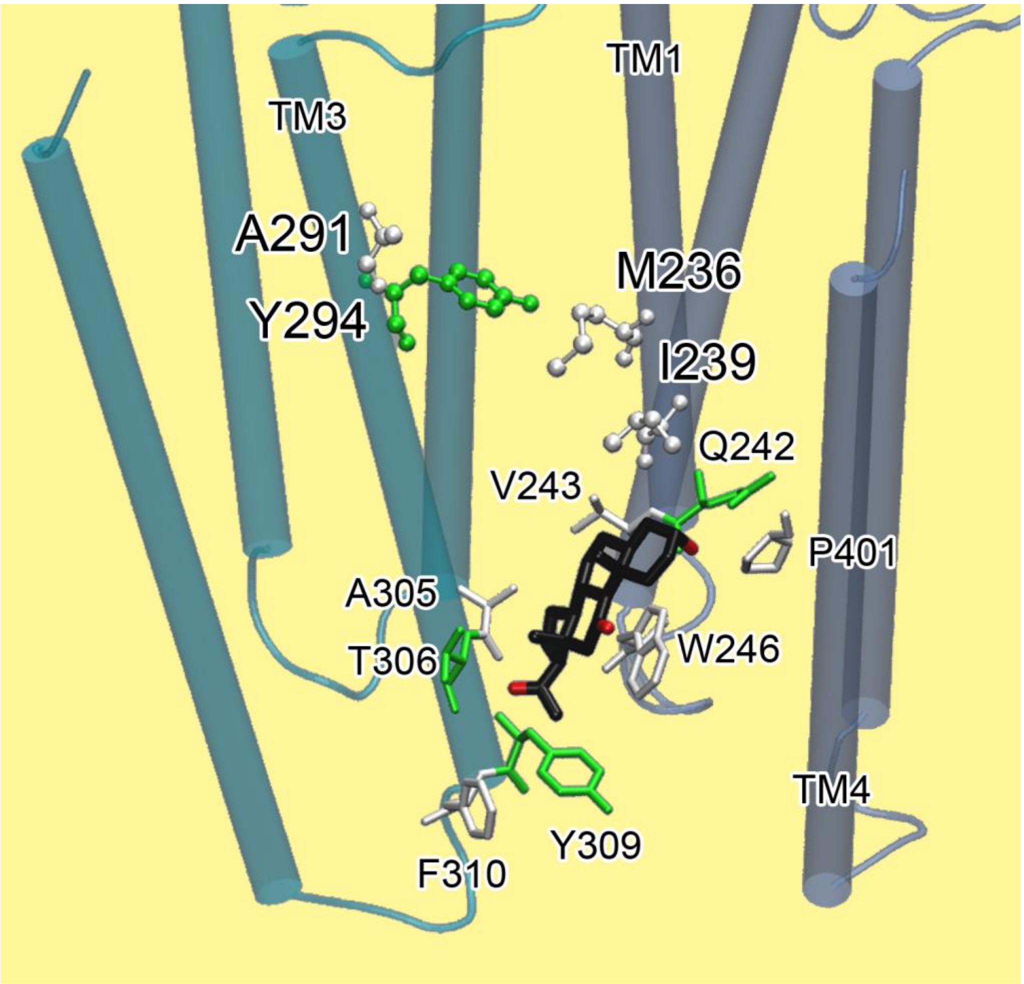

Supplementary Figure 6b. Alphaxalone binding site and its close proximity to propofol and etomidate binding sites identified previously by photolabeling. Residues of the α1GABA$_A$R chimera within 4 Å of alphaxalone (black sticks) are highlighted as sticks and labeled with a smaller font. Residues M236 and I239 were identified for the photolabeling of propofol[16], M236 for etomidate[17,18]. Residues A291 and Y294 were identified in the photolabeling of a barbiturate[19]. These residues are highlighted as CPK and labeled with a larger font. Hydrophobic residues are colored in white and polar residues are colored in green.

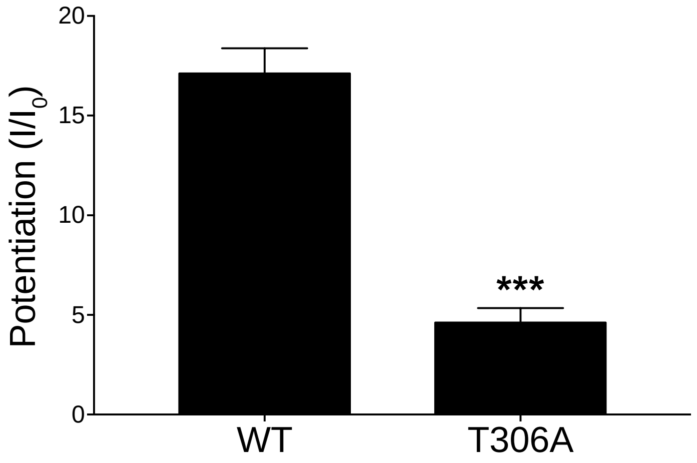

Supplementary Figure 7. Alphaxalone potentiation of α1β3GABA$_A$R. Alphaxalone potentiation was measured in *Xenopus* oocytes expressing full-length WT or T306A mutant α1β3GABA$_A$Rs. Data (I/I_0) were recorded at the EC_5 concentration of GABA with and without 3 μM alphaxalone. Error bars represent SEM ($n \geq 6$ oocytes). Statistical significance was assessed by the Student's t-test and asterisks indicate statistical difference from WT at $p < 0.001$ (***).

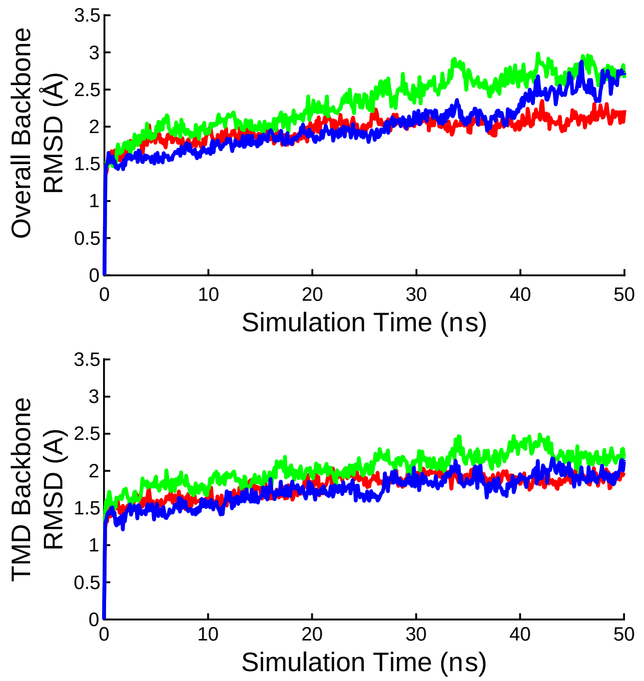

Supplementary Figure 8. Structural Equilibration of the alphaxalone-bound ELIC-α1GABA$_A$R in MD simulations. Backbone RMSD values over the course of three replicate 50-ns simulations are shown for all residues (top) and the TMD residues only (bottom). The protein, particularly the TMD, is stable in the MD simulations.

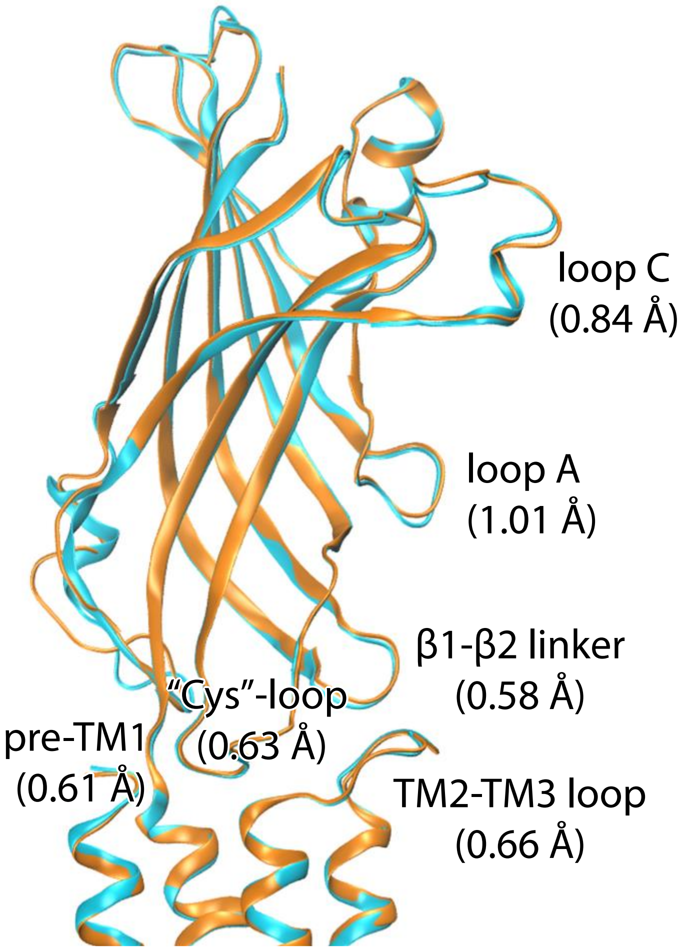

Supplementary Figure 9. **Structural changes in the ECD and ECD-TMD interface of the apo (orange) and alphaxalone-bound (cyan) ELIC-α1GABA$_A$R.** RMSDs are calculated for the marked regions based on the aligned the two structures.

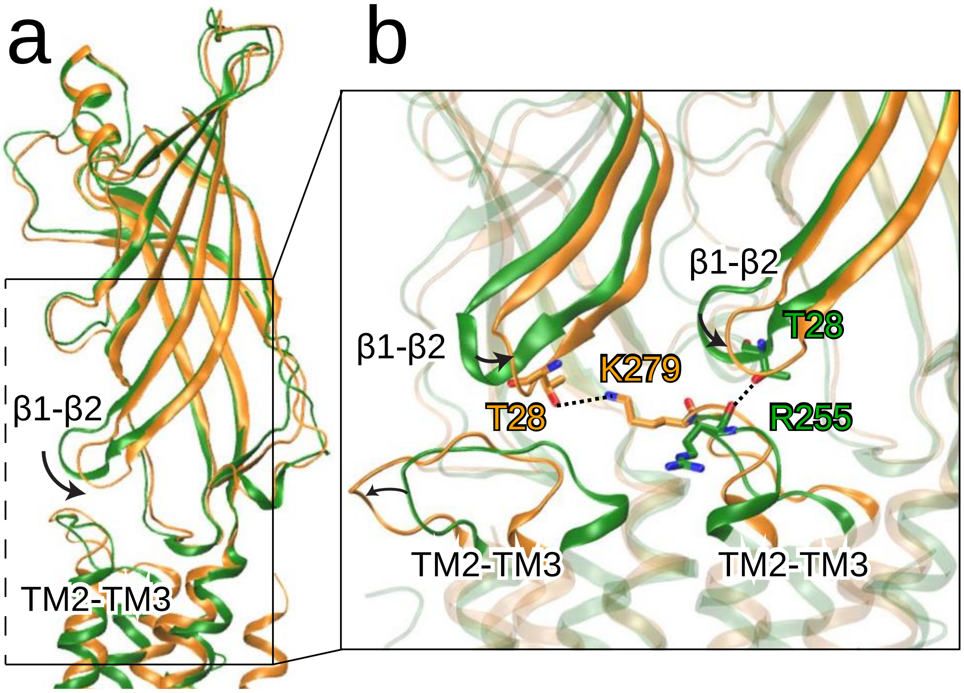

Supplementary Figure 10. Structural comparison at the ECD-TMD interface between apo ELIC-α1GABA$_A$R (orange) and apo ELIC (green). (**a**) Significant displacements of the TM2-TM3 loop and the β1-β2 linker shown in the structures of the apo ELIC-α1GABA$_A$R (orange) and apo ELIC (green), aligned by the common ECD residues (P11-R199). Only a single subunit is shown for clarity. (**b**) In apo ELIC-α1GABA$_A$R (orange), the conserved K279 in the TM2-TM3 loop has a polar contact with the β1-β2 linker (T28) in the adjacent subunit (dashed line, 4.0 Å), whereas in ELIC (green), the equivalent residue R255 only interacts with the β1-β2 in the same subunit (dashed line, 3.1 Å).

TM1 ← TM2

−2′ 2′ 6′ 9′ 13′ 17′ 20′

α1GABA$_{A}$R$_{HUMAN}$	245	FWLNRESVPARTVFGVTTVLTMTTLSISARNSLPK	279
α5GABA$_{A}$R$_{HUMAN}$	248	FWLNRESVPARTVFGVTTVLTMTTLSISARNSLPK	282
β3GABA$_{A}$R$_{HUMAN}$	240	FWINYDASAARVALGITTVLTMTTINTHLRETLPK	274
GluCl	276	FWFDRTAIPARVTLGVTTLLTMTAQSAGINSQLPP	310
5HT$_{3A}$R$_{MOUSE}$	242	FCLPPDSG-ERVSFKITLLLGYSVFLIIVSDTLPA	275
αnAChR$_{TORMA}$	233	FYLPTDSG-EKMTLSISVLLSLTVFLLVIVELIPS	266
βnAChR$_{TORMA}$	239	FYLPPDAG-EKMSLSISALLALTVFLLLLADKVPE	272
γnAChR$_{TORMA}$	240	YFLPAQAGGQKCTLSISVLLAQTIFLFLIAQKVPE	274
δnAChR$_{TORMA}$	247	FYLPAESG-EKMSTAICVLLAQAVFLLLTSQRLPE	280
GLIC	215	FWS--TSYEANVTLVVSTLIAHIAFNILVETNLPK	247
ELIC	222	FWL--ESFSERLQTSFTLMLTVVAYAFYTSNILPR	254
α1GlyR$_{HUMAN}$	242	FWINMDAAPARVGLGITTVLTMTTQSSGSRASLPK	276
α3GlyR$_{HUMAN}$	242	FWINMDAAPARVALGITTVLTMTTQSSGSRASLPK	276

Supplementary Figure 11. Sequence alignment of TM2 helices from selected pLGICs. Pore-lining residues are marked in blue from the corresponding structures in Supplementary Table 1 below. Note that residues in the 2′ and 13′ positions for the anion channel GluCl are more similar to those found in cation channels and significantly different from those in GABA$_{A}$R channels. In particular, the difference in hydrophobicity of the 2′ residues may explain why the pore of our resting α1GABA$_{A}$R chimera is most constricted at 2′ while the pore of the resting GluCl is most constricted at 9′.

Supplementary Table 1. Structures providing the sequences in Supplementary Fig. 11

Channel	State	PDB	Reference
Human α1GABA$_{A}$R	Apo	6D1S	Current study
	Desensitized	6CDU	Current study
		5OSA	[11]
Human α5GABA$_{A}$R	Desensitized	5OJM	[12]
Human β3GABA$_{A}$R	Desensitized	4COF	[6]
GluCl	Nanobody-bound	4TNV	[1]
Mouse 5HT$_{3A}$R	Apo	6BE1	[2]
	Nanobody-bound	4PIR	[3]
Torpedo nAChR	Apo	2BG9	[4]
GLIC	Apo	4NPQ	[5]
ELIC	Apo	3RQU	[10]
		2VL0	[20]
Human α1GlyR	Desensitized	3JAF	[8]
	Antagonist-bound	3JAD	
Human α3GlyR	Desensitized	5TIO	[7]

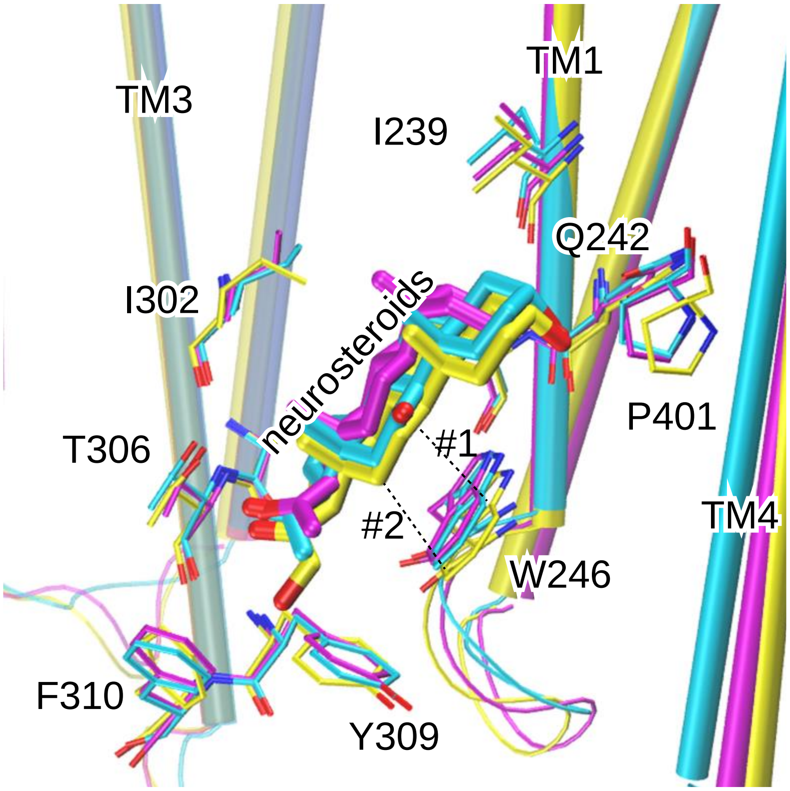

Supplementary Figure 12. A common neurosteroid-binding site in GABA$_A$Rs. Alphaxalone (cyan) binds to ELIC-α1GABA$_A$R at the same site as previously identified for the endogenous potentiating neurosteroids tetrahydrodeoxycorticosterone (THDOC, yellow) in GLIC-α1GABA$_A$R[11] and pregnanolone (purple) in β3GABA$_A$R-α5GABA$_A$R[12]. In spite of variations in some side-chain orientations, the same group of residues show close contact (4 Å) with neurosteroids among all three structures. Note that the marked residue numbers are based on ELIC-α1GABA$_A$R; residue numbering varies in different structures. The ring stacking interaction between neurosteroids and W246 (W249 in α5GABA$_A$R) was measured by the two distances (dashed lines) as defined in Fig. 5a in the main text. Distance #1 and #2 are 4.52 ± 0.07 Å and 3.93 ± 0.09 Å for alphaxalone, 4.61 ± 0.03 Å and 4.03 ± 0.08 Å for THDOC, and 4.87 ± 0.02 Å and 4.58 ± 0.03 Å for pregnanolone (mean ± SD).

Supplementary Table 2. Primers for ELIC-α1GABA$_A$R constructs.

Construct	Primers
ELIC-α1GABA$_A$R	5′-CGGATCGACGCCGTCAGGAAGATTGGCTACTTT-3′<br>5′-AAAGTAGCCAATCTTCCTGACGGCGTCGATCCG-3′
ICD Deletion	5′-GTAAACTATTTCACTAAGAGAGGCGTGGAGAGTGTCAGCAAAATTGACCGAC-3′<br>5′-GTCGGTCAATTTTGCTGACACTCTCCACGCCTCTCTTAGTGAAATAGTTTAC-3′
C-Terminus Truncation	5′-ACTGGGCTACGTATTTAAACTGACTCGAGCACCAC-3′<br>5′-GTGGTGCTCGAGTCAGTTTAAATACGTAGCCCAGT-3′
Q242L Mutant	5′-GACAGTGATTCTCTCACTAGTCTCCTTCTGGCTCA-3′<br>5′-TGAGCCAGAAGGAGACTAGTGAGAGAATCACTGTC-3′
W246L Mutant	5′-ATTCTCTCACAAGTCTCCTTCTTGCTCAACAGAGAG-3′<br>5′-CTCTCTGTTGAGCAAGAAGGAGACTTGTGAGAGAAT-3′
T306A Mutant	5′-CAGCTCTGATTGAGTTTGCCGCAGTAAACTATTTCACTAAGA-3′<br>5′-TCTTAGTGAAATAGTTTACTGCGGCAAACTCAATCAGAGCTG-3′

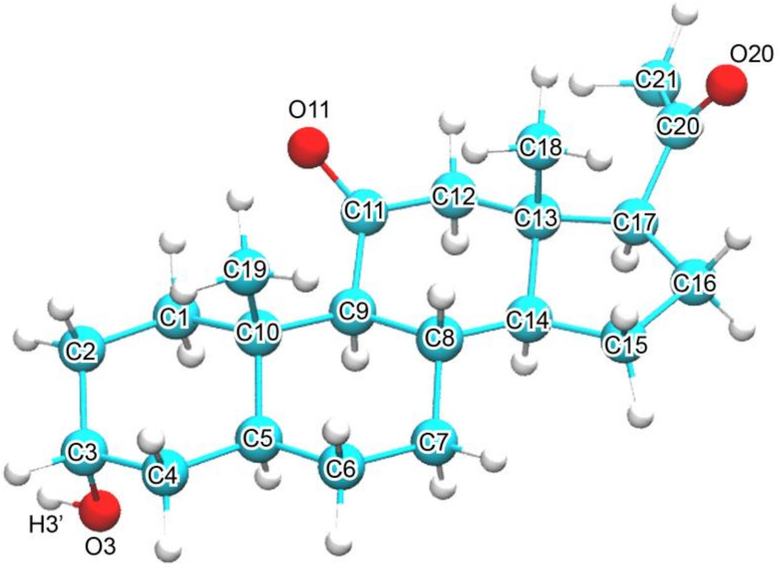

Supplementary Figure 13. The optimized structure of alphaxalone that was obtained using Gaussian16 software at the MP2/6-31G(d) level of theory.

Supplementary Table 3. Summary of Lennard–Jones and electrostatic parameters for alphaxalone.

Atom Name*	Charge (e)	ε (kcal/mol)	Rmin/2 (Å)
C1	-0.183	-0.056	2.01
C2	-0.180	-0.056	2.01
C3	0.138	-0.032	2.00
H3’	0.410	-0.046	0.23
O3	-0.651	-0.192	1.77
C4	-0.179	-0.056	2.01
C5	-0.089	-0.032	2.00
C6	-0.180	-0.056	2.01
C7	-0.180	-0.056	2.01
C8	-0.089	-0.032	2.00
C9	0.043	-0.032	2.00
C10	-0.014	-0.032	2.00
C11	0.463	-0.090	2.00
O11	-0.490	-0.050	1.70
C12	-0.140	-0.056	2.01
C13	0.001	-0.032	2.00
C14	-0.094	-0.032	2.00
C15	-0.180	-0.060	2.02
C16	-0.176	-0.060	2.02
C17	-0.483	-0.036	2.01
C18	-0.270	-0.078	2.05
C19	-0.272	-0.078	2.05
C20	0.754	-0.090	2.00
O20	-0.524	-0.050	1.70
C21	-0.229	-0.078	2.05
H3, H5, H8, H9, H14, H17 (×1)[#]	0.090	-0.045	1.34
H1, H2, H4, H6, H7, H12, H15, H16, H21 (×2)[#]	0.090	-0.035	1.34
H18, H19, H21 (×3)[#]	0.090	-0.024	1.34

*Atom names are labeled in Supplementary Figure 13. [#]Non-polar hydrogen atoms are named according to their corresponding carbon atom.

Supplementary Table 4. Comparison of molecular and quantum mechanical interaction energies and geometries for alphaxalone-water complexes. Quantum mechanical calculations of alphaxalone interactions with water molecules were performed using Gaussian16.

**Interaction***	**ΔE_{HF} (kcal/mol)**	**ΔE_{CGenFF} (kcal/mol)**	**$\Delta\Delta E$ (kcal/mol)**	**R_{HF} (Å)**	**R_{CGenFF} (Å)**	**ΔR (Å)**
H3'…OHH	-6.03	-6.30	-0.27	2.04	2.04	0.00
O3…HOH	-6.73	-7.07	-0.34	2.99	3.04	0.05
O11…HOH	-7.47	-7.29	0.18	2.97	2.87	-0.10
O20…HOH	-7.09	-6.46	0.63	2.98	2.88	-0.10
AD			0.05			-0.04
RMSD			0.39			0.07
AAD			0.36			0.06

*Atom names are labeled in Supplementary Figure 13.

Supplementary Table 5. Molecular and quantum mechanical dipole moments for alphaxalone.

**μ component (Debye)**	**HF/6-31G(d)**	**MP2/6-31G(d)**	**CGenFF**
D_x	1.570	1.410	2.122
D_y	0.604	0.346	-1.660
D_z	-2.911	-2.376	-3.105
D_{Total}	3.362	2.784	4.110

SUPPLEMENTARY REFERENCES

1. Althoff T, Hibbs RE, Banerjee S, Gouaux E. X-ray structures of GluCl in apo states reveal a gating mechanism of Cys-loop receptors. *Nature* **512**, 333-337 (2014).

2. Basak S, *et al.* Cryo-EM structure of 5-HT3A receptor in its resting conformation. *Nat Commun* **9**, 514 (2018).

3. Hassaine G, *et al.* X-ray structure of the mouse serotonin 5-HT3 receptor. *Nature* **512**, 276-281 (2014).

4. Unwin N. Refined structure of the nicotinic acetylcholine receptor at 4A resolution. *J Mol Biol* **346**, 967-989 (2005).

5. Sauguet L, *et al.* Crystal structures of a pentameric ligand-gated ion channel provide a mechanism for activation. *Proc Natl Acad Sci U S A* **111**, 966-971 (2014).

6. Miller PS, Aricescu AR. Crystal structure of a human GABAA receptor. *Nature* **512**, 270-275 (2014).

7. Huang X, Chen H, Shaffer PL. Crystal Structures of Human GlyRalpha3 Bound to Ivermectin. *Structure* **25**, 945-950 e942 (2017).

8. Du J, Lu W, Wu S, Cheng Y, Gouaux E. Glycine receptor mechanism elucidated by electron cryo-microscopy. *Nature* **526**, 224-229 (2015).

9. Morales-Perez CL, Noviello CM, Hibbs RE. X-ray structure of the human alpha4beta2 nicotinic receptor. *Nature* **538**, 411-415 (2016).

10. Pan J, *et al.* Structure of the pentameric ligand-gated ion channel ELIC cocrystallized with its competitive antagonist acetylcholine. *Nat Commun* **3**, 714 (2012).

11. Laverty D, *et al.* Crystal structures of a GABAA-receptor chimera reveal new endogenous neurosteroid-binding sites. *Nat Struct Mol Biol* **24**, 977-985 (2017).

12. Miller PS, *et al.* Structural basis for GABAA receptor potentiation by neurosteroids. *Nat Struct Mol Biol* **24**, 986-992 (2017).

13. Nury H, *et al.* One-microsecond molecular dynamics simulation of channel gating in a nicotinic receptor homologue. *Proc Natl Acad Sci U S A* **107**, 6275-6280 (2010).

14. Mowrey D, *et al.* Asymmetric ligand binding facilitates conformational transitions in pentameric ligand-gated ion channels. *J Am Chem Soc* **135**, 2172-2180 (2013).

15. Willenbring D, Liu LT, Mowrey D, Xu Y, Tang P. Isoflurane alters the structure and dynamics of GLIC. *Biophys J* **101**, 1905-1912 (2011).

16. Jayakar SS, *et al.* Multiple propofol-binding sites in a gamma-aminobutyric acid type A receptor (GABAAR) identified using a photoreactive propofol analog. *The Journal of biological chemistry* **289**, 27456-27468 (2014).

17. Li GD, Chiara DC, Sawyer GW, Husain SS, Olsen RW, Cohen JB. Identification of a GABAA receptor anesthetic binding site at subunit interfaces by photolabeling with an etomidate analog. *J Neurosci* **26**, 11599-11605 (2006).

18. Chiara DC, Dostalova Z, Jayakar SS, Zhou X, Miller KW, Cohen JB. Mapping general anesthetic binding site(s) in human alpha1beta3 gamma-aminobutyric acid type A receptors with [(3)H]TDBzl-etomidate, a photoreactive etomidate analogue. *Biochemistry* **51**, 836-847 (2012).

19. Chiara DC, *et al.* Specificity of intersubunit general anesthetic-binding sites in the transmembrane domain of the human alpha1beta3gamma2 gamma-aminobutyric acid type A (GABAA) receptor. *The Journal of biological chemistry* **288**, 19343-19357 (2013).

20. Hilf RJ, Dutzler R. X-ray structure of a prokaryotic pentameric ligand-gated ion channel. *Nature* **452**, 375-379 (2008).
